# Impact of High-Dose Supplemental Paprika Extract Feeding on Egg Storage and Biochemical Parameters in Laying Hens

**DOI:** 10.3390/ani14192856

**Published:** 2024-10-04

**Authors:** Sadao Kojima

**Affiliations:** Animal Science Division, Tokyo Metropolitan Agriculture and Forestry Research Center, 6-7-1 Shinmachi, Ome, Tokyo 198-0024, Japan; sadao-kojima@tdfaff.com; Tel.: +81-428-31-2171

**Keywords:** paprika extract, rhode island red, silky fowl, storage, biochemical, egg quality

## Abstract

**Simple Summary:**

This study explored how adding paprika extract to the diet of laying hens affects egg quality and their blood health. We tested two types of hens, Rhode Island Red and Silky Fowl, by giving them either their usual feed or the same feed with paprika extract for 28 days. We stored the eggs at two different temperatures to see how the paprika might influence their quality over time. We found that paprika improved the color of the egg yolks and affected the hens’ blood cholesterol levels in different ways depending on the breed. For Silky Fowl hens, more paprika led to higher levels of good cholesterol, while this was not the case for Rhode Island Red hens. Overall, our findings suggest that paprika extract can improve egg quality and influence blood cholesterol, but the effects vary between different breeds of hens. This information could help farmers enhance egg production and nutritional value through dietary adjustments.

**Abstract:**

This study aimed to evaluate the effects of paprika extract supplementation on egg storage and blood biochemical parameters in 63-week-old Rhode Island Red (RIR) and Silky Fowl (SF) hens. The hens were divided into three groups: a control group with a basal diet and two groups receiving the basal diet with paprika extract. The trial lasted 28 days, with egg quality (yolk color and albumen pH) assessed after storing eggs at 25 °C for 21 days. A total of 144 eggs were used in 42 treatments (two breeds, three diets, and seven storage periods) with three eggs examined each (four eggs were used on day 0 and five eggs on day 21). Additionally, the yolk carotenoid content, yolk color, and pH of eggs stored at 4 °C and 25 °C were compared. Results showed that yolk color fan score (YCFS) decreased with storage, and SF had a higher albumen pH than RIR, with both breeds exhibiting an increase in pH over time. HDL cholesterol (HDL-C) levels and the HDL-C to total cholesterol ratio were significantly influenced by breed, diet, and their interaction. The HDL-C level in SF was affected linearly and quadratically by diet, while no such trend was observed in RIR. The study concluded that paprika extract affects egg quality and blood lipid profiles differently in different breeds, highlighting breed-specific responses to dietary supplementation.

## 1. Introduction

Yolk color, influenced by carotenoids in the feed, is a critical aspect of egg quality, with a preferred range of 10–14 on the Roche Yolk Color Fan (RYCF) in some European and Asian nations [1]. The RYCF score correlates with yolk carotenoid content [2]. Beyond its visual appeal to consumers, yolk color plays a significant role, inhibiting yolk lipid oxidation and enhancing singlet oxygen-quenching activity [3,4,5]. Furthermore, natural and synthetic pigments have been used to obtain specific egg yolk colors [6,7]. However, some countries, such as Sweden, do not allow the use of synthetic pigments [6]. Marigold flower and red pepper paprika extracts have been used as natural pigments to supplement laying hen diets [7]. Supplementation of paprika (*Capsicum annuum*) extract to laying hens has been reported to be effective in enhancing yolk color, especially redness (a* value) [8]. The feeding of paprika to laying hens has been shown to affect the blood lipid profile [9], and the feeding of red pepper has also been reported to reduce blood total cholesterol [10].

Silky Fowl (SF, *Gallus gallus domesticus*), originating from China, presents unique characteristics, including silky feathers, black-colored bones, and dark bluish skin. SF exhibits distinct metabolic features, with melanin deposits and pigment granules in various organs [11]. Traditional Chinese medicine attributes health benefits to SF meat and eggs, particularly for women and those with lung ailments [12]. The metabolism of the SF hen is different from that of other chickens [13]. The Rhode Island Red (RIR) hen is a common chicken breed with a high egg-laying rate. Research by Kojima et al. [5] showed that high levels of carotenoids in the diet can improve the yolk color of hen’s eggs. Moreover, they revealed breed-specific variations in yolk carotenoid content, with SF hens accumulating more lutein and zeaxanthin in their yolks compared to RIR hens.

There are many reports on the effect of storage of chicken eggs on yolk color. It has been reported that the storage period of eggs affects yolk color [14,15,16], while there is another report that the storage period does not affects yolk color [17,18,19]. However, there are few reports on the effects of different storage temperatures on yolk color [14]. There have been reports on the effects of dietary carotenoids on egg quality and blood biochemical parameters [3,19,20]. However, there have been no reports on the effects of breeds. While dietary carotenoids are known to improve the color of egg yolks [21], the effects of different breeds on the preservation of carotenoid-enriched eggs and the biochemical value of laying hens have not been fully investigated. To our knowledge, there are no reports on paprika extracts that have studied all three. Based on the previous reports [5,13], we hypothesized that SF would show a breed-specific response to the added feeding of paprika extract. Therefore, for studying these three, in this study, we investigated the effect of the storage of eggs produced by feeding paprika extract to laying hens on egg quality and blood biochemical values using RIR and SF breeds.

## 2. Materials and Methods

### 2.1. Experimental Design, Diets, and Husbandry

All experimental procedures were conducted following the guidelines for animal experiments of the Tokyo Metropolitan Agriculture and Forestry Research Center. Sixty-three-week-old laying hens (48 RIR and 48 SF) were utilized and placed in wire-floored group cages into 3 dietary treatments with 2 replicates of 8 hens each. Before the trial start, all laying hens were fed with a basal diet in mash form for 2 weeks (Table 1, Power Layer-177, JA Higashinihon Kumiai Shiryou, Ota, Japan).

The hens were fed the basal diet (control group), the basal diet plus 0.6% dietary paprika extract (PA1 group, basal diet with 30 mg/kg paprika extract carotenoids), or the basal diet plus 1.2% dietary paprika extract (PA2 group, basal diet with 60 mg/kg paprika extract carotenoids). Paprika extract was commercially available in powdered form (Ran-Red^®^, Elanco Japan k.k., Tokyo, Japan; including 5 g/kg carotenoid). The carotenoid content of PA1 was based on a previous report [5], and double the amount was used in PA2, which corresponds to the high-dose group. The feeding experiment lasted 28 days, and food and water were provided ad libitum during the experimental period. All hens were placed in a windowless poultry house and the cages were double-decker rows. Each cage had the following dimensions: width of 241 cm, depth 95 cm, and a minimum height 70 cm. One quarter of each of its cages is a nesting area was separated by a red curtain composed of plastic strips. Lighting regime applied during the experiment was 16 h of light and 8 h of darkness. The average room temperature was maintained at about 22 °C.

### 2.2. Sample Preparation

Eggs from the 4th week of the experimental period were utilized for the measurements. Shell eggs were stored in an incubator set at 25 °C and observed for 21 days. Eggs were broken on days 0, 1, 2, 3, 7, 14, and 21 to investigate egg yolk color and albumen pH. The total 144 eggs were used in 42 treatments (2 breeds × 3 diets × 7 storage periods) with 3 eggs examined each (4 eggs were used on day 0 and 5 eggs on day 21). Day 0 eggs were broken and measured within 12 h of being laid. Additionally, fresh eggs were stored at 4 °C and 25 °C for 21 days. All eggs were preserved by placing them on plastic trays with the small end down. The egg yolk color, total carotenoid content in the yolk, and pH of the albumens and yolks were measured of 12 eggs per treatment.

### 2.3. Measurement of Yolk Color

Yolk color was determined using the DSM Yolk Color Fan (DSM Nutritional Products, Basel, Switzerland) score (1–16), and it was set to 17 when the score exceeded 16. Egg yolk color was measured using a spectrophotometer (CM-600d, Konica Minolta, Tokyo, Japan) and reported in the CIELAB system to calculate the values of lightness (L*), redness (a*), and yellowness (b*), and the ratio of redness to yellowness (a/b).

### 2.4. pH Measurement

After the eggs were carefully broken, the albumen was separated from the egg yolk using a household egg separator. The pH values of the albumen and yolk were measured using a pH meter (D-72, Horiba Advanced Techno Co., Ltd., Kyoto, Japan).

### 2.5. Blood Collection and Blood Biochemistry Test

At the end of the experiment, a total of 5 birds per replicate were randomly selected without fasting. Blood samples were collected from the wing vein into serum separator tubes without an anticoagulant agent. Blood samples were centrifuged at 1630× *g* for 10 min at 4 °C to separate the serum. Then, the serum samples were frozen at −80 °C until analysis. Total cholesterol (T-Chol), HDL cholesterol (HDL-C), and triglyceride (TG) levels in the serum were measured using an automatic dry chemistry analyzer (FUJI DRI-CHEM NX700iV, Fujifilm, Tokyo, Japan), according to the manufacturer’s instructions. The T-Chol minus HDL-C (non-HDL) and T-Chol to HDL-C ratio (HDL/T) were calculated.

### 2.6. Total Carotenoid Content in Yolk

Yolks of eggs laid after 4 weeks of treatment were utilized to analyze the total carotenoid content. The total carotenoid content in the yolks of raw stored eggs was measured by a UV-visible spectrophotometer (UVmini-1240, Shimadzu, Kyoto, Japan). For each egg, 1.5 g of yolk was weighed in a centrifuge tube and vigorously shaken with acetone (30 mL). After homogenization, the extract was transferred to a brown flask. Subsequently, 30 mL of acetone was added to the residue and allowed to stand after homogenization. This process was repeated 3 times, and each extract was transferred to a brown measuring flask. Acetone was added to the measuring flask to make up the volume to 100 mL. The resulting extract was filtered through a 0.45 µm Millipore filter disk (Merck KGaA, Darmstadt, Germany) and used as the test solution. Carotenoid content was calculated using the absorbance at 453 nm, and the total quantity of carotenoids was assessed using an optical density of λmax = 450 to 470 nm. For quantification, the following extinction coefficients, absorbance of 1% concentration, were adopted: 2400 for the control; 2072 for the PA groups [22].

### 2.7. Statistical Analysis

All statistical analyses were carried out using R software version 3.6.2 (accessed on 12 December 2019, http://www.R-project.org/) [23]. Data concerning the egg storage period were analyzed using a repeated three-way ANOVA, and the interactions among breed, diet, and storage period were examined. Two-way ANOVA was performed for blood biochemical values and egg storage temperatures. Significant differences among various treatments were determined using the Tukey’s HSD (Honestly Significant Difference) test. Additionally, linear and quadratic regression analyses were used for blood biochemistry values as well as changes in egg yolk color during storage. Statistical significance was defined at *p* < 0.05.

## 3. Results

### 3.1. Yolk Color and Albumen pH of Raw Eggs at 25 °C

The impact of breed, diet, and storage period on the yolk color fan score (YCFS) in raw eggs stored at 25 °C is presented in Table 2. Significant effects were observed for breed, diet, and storage period (*p* < 0.001). Notably, significant interactions between breed and diet (*p* = 0.006) and diet and storage period (*p* = 0.019) were noted. Storage duration of eggs fed paprika extract at 25 °C also affected egg yolk color linearly and quadratically (*p* < 0.05). In contrast, there was no significant linear change in yolk color in the control group.

Albumen pH in raw eggs stored at 25 °C was monitored during a 21-day storage period (Table 3). Significant main effects were observed for breed and storage period (*p* < 0.001), while no significant effect of diet was identified (*p* = 0.360). Notably, significant interactions between breed and storage period (*p* < 0.001) and breed, diet, and storage period (*p* = 0.016) were observed. As the storage period increased, albumen pH increased linearly and quadratically in all treatments (*p* < 0.001).

### 3.2. Blood Llipid Metabolisms

Table 4 displays the blood biochemical results. The effect of breed was significant for four parameters (HDL-C, TG, non-HDL, and HDL/T; *p* < 0.001), except for T-Chol (*p* > 0.05). HDL-C and HDL/T demonstrated a significant effect of diet (*p* = 0.013 and *p* = 0.006, respectively) and a significant interaction between breed and diet (*p* = 0.002 for both). TG levels in the SF decreased with increasing dietary carotenoid content, showing a significant difference between the control and PA2 groups (*p* < 0.05). However, RIR did not exhibit a similar trend. HDL-C and HDL/T in the SF were increased with increasing dietary carotenoid content, showing a significant difference between the control and PA2 groups (*p* < 0.05). In the SF, HDL-C and HDL/T were affected linearly and quadratically by the diet (*p* < 0.01). On the other hand, no significant effects were observed for RIR with any parameters, except for the quadratic effect on HDL/T (*p* = 0.034).

### 3.3. Yolk Carotenoid Content, Yolk Color, and pH of Raw Eggs at 4 °C and 25 °C

As outlined in Table 5, significant main effects of breed were observed on yolk carotenoid content, YCFS, L* values, b* values, a/b values, and albumen pH in raw eggs stored at 4 °C and 25 °C for 21 days (*p* = 0.002 for YCFS, *p* < 0.001 for other items). There was a significant main effect of storage temperature on yolk carotenoid content (*p* = 0.007), YCFS (*p* < 0.001), L* values (*p* < 0.001), albumen pH (*p* < 0.001), and yolk pH (*p* = 0.006) of raw eggs stored at 4 °C or 25 °C for 3 weeks. YCFS decreased significantly at 25 °C compared to 4 °C in both breeds (*p* < 0.05), and the L* values of SF were significantly higher at 25 °C than at 4 °C (*p* < 0.05), with no significant difference in RIR (*p* > 0.05). The albumen pH of RIR was significantly higher at 25 °C than at 4 °C (*p* < 0.05), while there was no significant difference in SF (*p* > 0.05). An interaction between breed and storage temperature for albumen pH was deemed significant (*p* < 0.001). This is attributed to the fact that the changes of albumen pH of SF and RIR by storage temperature are not parallel.

## 4. Discussion

In this investigation, we delved into the impact of paprika extract supplementation on egg storage and blood biochemistry. Yolk color is an important factor of internal quality of eggs. Paprika extract is a feed additive that effectively enhances egg yolk color even at a low addition level of about 0.1% [24,25]. Our study showed that the YCFS of eggs stored at 25 °C exhibited a period-dependent decrease, deviating from previous findings [18,19,26]. Our results showed that through the 21-day observation at 25 °C, a noteworthy linear and quadratic regression was identified in both breeds fed with paprika extract, while no significant linear regression observed in the control group. The interaction between breed and diet was ascribed to a more pronounced increase in YCFS in RIR than in SF with escalating paprika extract amounts. Thus, RIR exerted a more substantial effect on YCFS than SF with PA supplementation at high concentrations, such as 60 mg/kg (PA2). The significant interplay between diet and storage period stemmed from a more considerable reduction in YCFS scores in the PA group compared to the control group. The impact of storage duration at 25 °C on the reduction of YCFS is more pronounced for dark-colored egg yolks. A previous study where laying hens were fed astaxanthin, Wang et al. [27] noted a decrease in CIELAB levels and carotenoid content (astaxanthin and zeaxanthin) in egg yolks from raw eggs stored at 4 °C for 28 days. This aligns with the report by Islam et al. [2] suggesting a regression between the total carotenoid content in egg yolk and YCFS, alongside yolk CIELAB values. Conversely, no change in YCFS was detected in raw eggs enriched with lutein content stored at 4 °C for 28 days [28]. When laying hens were fed marigold extract, the YCFS of raw eggs stored at 4 °C for 28 days surpassed that of fresh eggs [26]. Our study unveiled a linear and quadratic decrease in the YCFS values of eggs enriched with paprika extract during storage at 25 °C. While pigments from paprika extract have a robust coloring effect on egg yolk [29], their sustainability may vary based on storage temperature and duration.

Albumen pH serves as a valuable gauge for assessing changes in albumen quality during storage [30]. In previous studies, with increasing shelf life and storage temperature, albumen pH tends to rise [19,31,32,33]. Similarly, in this investigation, the primary effects of albumen pH and storage period were statistically significant (*p* < 0.001). Across both breeds, albumen pH exhibited an incremental pattern correlating significantly with the duration of storage, and notably, the pH of SF consistently surpassed that of RIR throughout this storage period. Specifically, on day 0 (laying day), the albumen pH in SF was more than 0.7 units higher than in RIR. The present study showed that the variance in albumen pH among breeds at the time of laying is sustained throughout the storage period at 25 °C. The notable interaction between breed and storage period is attributed to a substantial spike observed in RIR within the initial 24 h at 25 °C. Furthermore, the significant interplay among breed, diet, and storage period may be linked to a more pronounced increase in pH at day 21 in PA1 of RIR and the control group of SF. A more extended period of observation is warranted to ascertain the true significance of this interaction.

HDL, a crucial lipoprotein, plays a significant role in transporting carotenoids into the bloodstream [34]. Patients with Alzheimer’s disease, vascular comorbidities, and risk factors (such as the elevated intima-media thickness of the common carotid artery and/or type 2 diabetes mellitus) exhibited markedly lower concentrations of plasma carotenoids (lutein, zeaxanthin, and lycopene) and HDL-C compared to control subjects without cognitive impairment or vascular issues [35]. Notably, dietary supplementation with astaxanthin led to a substantial increase in plasma HDL-C levels and a concurrent decrease in TG content in laying hens [3]. However, conflicting findings exist regarding the impact of dietary astaxanthin on blood lipid metabolism [20]. Notably, these studies utilized the same line of Hy-Line Brown layers, differing primarily in the age of the birds (20 and 50 weeks, respectively). Ko et al. [36] observed a significant decrease in the carotenoid content (lutein and zeaxanthin) in egg yolks with aging in laying hens, proposing that the elevated carotenoid content in eggs laid by early-age hens results from active metabolism. Conversely, Marzec et al. [17] reported in a study involving ISA Brown Hens that age did not influence yolk color. In Red-legged Partridges (*Alectoris rufa*), Bortolotti et al. [37] discovered that early in the laying season, plasma and yolk carotenoids varied with diet and were correlated with one another. In the same report, late in the season, a dietary effect was evident only for yolks, and there was no relationship between plasma and egg levels of individual hens. In our study, 63-week-old laying hens were utilized, which was in the late laying season. The findings indicated that RIR did not exert an impact on blood lipid metabolism, consistent with prior reports [20]. In contrast, SF demonstrated an increase in HDL-C and a decrease in TG levels, mirroring the effects observed in hens at 20 weeks [3]. Our biochemical regressions highlighted changes in HDL-C, non-HDL, and HDL/T in the SF contingent on dietary carotenoid levels. A similar result was observed in the previous study that was also a significant increase in HDL-C levels in the plasma of mice fed astaxanthin [38]. While no significant regression was found between TG levels in SF serum and dietary carotenoid concentrations, TG levels in SF serum tended to decrease depending on the carotenoid content in the diet. Importantly, these blood biochemistry changes were evident in the SF but not in the RIR in this study. These results suggest that SF, as a specific breed, responds to dietary carotenoids affecting blood lipid metabolism throughout the laying period. Furthermore, SF may be more susceptible to dietary carotenoids than other laying hens, especially in the early laying season, but further research is needed to clarify this.

In our study, variations in yolk carotenoid content, yolk color, and pH were noted in raw eggs enriched with carotenoids from paprika extract stored at 4 °C and 25 °C for 21 days. The carotenoid levels in egg yolks may significantly decrease during storage [34]. Notably, both astaxanthin and zeaxanthin contents in egg yolks decrease with prolonged storage [27]. When raw eggs enriched with paprika extract were stored at 4 °C and 25 °C for 21 days in our study, a significant main effect of storage temperature on total carotenoid content was observed (*p* < 0.007), indicating that the decline in carotenoid levels in egg yolks was influenced by the storage period and temperature.

The reduction in YCFS is impacted by the storage temperature and duration [14]. The YCFS of fresh eggs and eggs stored at 4 °C for 28 days was significantly affected by storage (*p* < 0.05) [16]. Conversely, there are reports suggesting that the YCFS does not decrease even after storage at 4 °C for more than 30 days, leading to inconsistency [19,39]. A decrease in the YCFS has been documented after storage at 20 °C [39] and approximately 35 °C [15]. In our study using paprika extract, the YCFS of eggs stored at 25 °C was significantly lower than that of eggs stored at 4 °C in both breeds (*p* < 0.05), and the main effect of storage temperature was also significant (*p* < 0.001). In a study by Grčević et al. [26] utilizing marigold extract, the YCFS of eggs stored at 4 °C for 28 days increased significantly (*p* < 0.05). In a report by Kralik et al. [28] comparing the YCFS in fresh eggs with that of eggs stored at 4 °C for 28 days, the YCFS was significantly reduced in conventional eggs but not in lutein-enriched eggs. These results suggest that different sources of pigments influence the shelf life of YCFS.

Dietary astaxanthin decreased the CIELAB values of egg yolks after 28 days of storage at 4 °C [27]. In the study using marigold extract, the CIELAB values of egg yolk increased after 28 days of storage at 4 °C [26]. In our study using paprika extract, lightness of yolk increased in both breeds at 25 °C compared to 4 °C, whereas redness showed the opposite response in RIR and SF. Moreover, the main effects of breed on lightness and yellowness were significant (*p* < 0.001). These results indicate that the response of the CIELAB values of egg yolk to raw egg storage is influenced not only by the source of carotenoids but also by the breed of laying hens.

According to previous reports, albumen pH increases during storage in a period-dependent manner [31,32], and the higher the storage temperature, the faster the increase in albumen pH [14,33]. Our results support these findings. SF exhibited a slower increase in albumen pH than RIR, and there was an interaction between breed and storage temperature. The cause of the increase in albumen pH is known to be alkalinization due to the release of carbon dioxide from albumen [40]. Albumen pH increased during storage; a high storage temperature acted as a catalyst for this increase, and lowering the pH slows down the rate of albumen liquefaction, thereby maintaining albumen quality [30]. Significant main effects of storage period have been reported for increasing yolk pH [26,28]. However, there is a report that egg yolk pH increases depending on the storage period [19,32,40], while there is also a report that the storage temperature had no effect [14,18]. A previous study showed that the yolk weight is increased by the diffusion of water from the albumen [40]. However, the reason for this difference could not be fully explained by the available literature. In our study, there was a significant effect of storage temperature, and the change in yolk pH was smaller in SF than in RIR.

## 5. Conclusions

The development of carotenoid-enriched eggs has garnered attention due to their multifunctional biological properties [34]. In our prior investigation, we identified higher levels of lutein and zeaxanthin in egg yolks from SF hens compared to RIR [5]. In the present study, we observed that dietary carotenoids positively influenced blood lipid balance in SF. The distinctions in blood characteristics, particularly blood lipid levels, between SF and RIR suggest that SF may exhibit specific effects on blood lipid metabolism when supplemented with paprika extract. This study concluded that paprika extract affects egg quality and blood lipid profiles differently in different breeds, highlighting breed-specific responses to dietary supplementation.

## Figures and Tables

**Table 1:** Ingredients and nutrient levels of the basal diet ^1^ (air-dried basis).

Ingredients ^2^	Content [%]
Corn, dehulled rice, grain sorghum	55.0
Soybean meal, rapeseed meal	25.0
Rice bran, DDGS ^3^, wheat bran	6.0
Fish meal	1.0
CaCO_3_, tallow, CaHPO_4_, salt, paprika extract, SiO_2_	13.0
Nutrient Levels	
Crude protein	17.0
Crude fat	2.5
Crude fiber	5.0
Crude ash	14.5
Calcium	2.80
Phosphorus	0.45
ME, kcal/kg	2800

^1^ Commercial formula feed. ^2^ Ingredients and nutrient levels were according to the manufacturer’s label. ^3^ Corn distillers dried grains with solubles.

**Table 2:** Effect of storage period at 25 °C on egg yolk color ^1^ of eggs fed paprika extract (PA).

		Storage Period							*p*-Value	
Breed ^2^	Diet ^3^	Day 0	Day 1	Day 2	Day 3	Day 7	Day 14	Day 21	Linear	Quadratic
RIR	Cont	10.3 ^c^	10.0 ^d^	9.7 ^c^	9.0 ^b^	10.0 ^b^	9.0 ^b^	9.6 ^c^	0.193	0.120
	PA1	15.8 ^ab^	16.3 ^ab^	15.7 ^ab^	14.3 ^a^	14.3 ^a^	15.0 ^a^	14.0 ^ab^	0.003	0.008
	PA2	17.0 ^a^	16.7 ^a^	16.3 ^a^	16.0 ^a^	15.7 ^a^	15.3 ^a^	15.4 ^a^	0.004	0.004
SF	Cont	10.0 ^c^	9.0 ^d^	9.0 ^c^	9.0 ^b^	9.0 ^b^	8.7 ^b^	9.4 ^c^	0.652	0.022
	PA1	14.8 ^b^	14.3 ^c^	15.3 ^ab^	14.3 ^a^	14.0 ^a^	13.7 ^a^	13.4 ^b^	0.006	0.024
	PA2	16.3 ^ab^	15.3 ^bc^	14.3 ^b^	14.0 ^a^	14.3 ^a^	14.3 ^a^	13.8 ^ab^	0.007	0.009
Pooled SEM		0.60	0.74	0.72	0.69	0.62	0.69	0.45		
Source of variation			*p*-Value							
Breed			<0.001							
Diet			<0.001							
Period			<0.001							
Breed × Diet			0.006							
Breed × Period			0.706							
Diet × Period			0.019							
Breed × Diet × Period			0.491							

^a–d^ Means within a column with different letters differ significantly (*p* < 0.05). The numbers of data points were as follows: four for 0 days, three for 1, 2, 3, 7, and 14 days, and five for 21 days. ^1^ The yolk color was determined using the DSM-YCF score (1–16) and was set to 17 when the score exceeded 16. ^2^ RIR = Rhode Island Red; SF = Silky Fowl. ^3^ Cont = basal diet; PA1 = basal diet with 30 mg/kg paprika extract carotenoids; PA2 = basal diet with 60 mg/kg paprika extract carotenoids.

**Table 3:** Effect of storage period at 25 °C on the pH of albumen fed paprika extract (PA).

		Storage Period							*p*-Value	
Breed ^1^	Diet ^2^	Day 0	Day 1	Day 2	Day 3	Day 7	Day 14	Day 21	Linear	Quadratic
RIR	Cont	7.73 ^b^	8.83	9.26 ^ab^	9.14 ^d^	9.40 ^b^	9.45 ^b^	9.52 ^e^	<0.001	<0.001
	PA1	7.80 ^b^	8.92	9.11 ^ab^	9.27 ^c^	9.38 ^b^	9.47 ^b^	9.66 ^c^	<0.001	<0.001
	PA2	7.80 ^b^	8.88	8.93 ^b^	9.28 ^bc^	9.44 ^b^	9.48 ^b^	9.61 ^d^	<0.001	<0.001
SF	Cont	8.52 ^a^	8.96	9.44 ^ab^	9.38 ^ab^	9.48 ^ab^	9.60 ^a^	9.81 ^a^	<0.001	<0.001
	PA1	8.52 ^a^	8.98	9.50 ^a^	9.43 ^a^	9.56 ^a^	9.62 ^a^	9.68 ^b^	<0.001	<0.001
	PA2	8.53 ^a^	9.06	9.43 ^ab^	9.44 ^a^	9.56 ^a^	9.60 ^a^	9.68 ^bc^	<0.001	<0.001
Pooled SEM		0.08	0.03	0.06	0.03	0.02	0.02	0.02		
Source of variation			*p*-Value							
Breed			<0.001							
Diet			0.360							
Period			<0.001							
Breed × Diet			0.405							
Breed × Period			<0.001							
Diet × Period			0.075							
Breed × Diet × Period			0.016							

^a–e^ Means within a column with different letters differ significantly (*p* < 0.05). The numbers of data points were as follows: four for 0 days, three for 1, 2, 3, 7, and 14 days, and five for 21 days. ^1^ RIR = Rhode Island Red; SF = Silky Fowl. ^2^ Cont = basal diet; PA1 = basal diet with 30 mg/kg paprika extract carotenoids; PA2 = basal diet with 60 mg/kg paprika extract carotenoids.

**Table 4:** Effect of feeding paprika extract (PA) on biochemical values of laying hens.

Breed ^1^	Diet ^2^	T-Chol	HDL-C	TG	non-HDL	HDL/T
		mg/dL	mg/dL	mg/dL	mg/dL	Ratio
RIR	Cont	148.6	44.5 ^b^	2293.8 ^a^	104.1 ^a^	0.31 ^c^
	PA1	135.9	49.5 ^b^	2072.2 ^a^	86.4 ^ab^	0.37 ^bc^
	PA2	142.3	43.2 ^b^	2162.1 ^a^	99.1 ^a^	0.31 ^c^
	SEM	6.0	1.5	149.7	5.3	0.01
	Linear	0.676	0.725	0.726	0.708	0.940
	Quadratic	0.703	0.184	0.841	0.387	0.034
SF	Cont	136.0	54.2 ^b^	1549.9 ^a^	81.8 ^ab^	0.42 ^bc^
	PA1	129.9	59.9 ^b^	1090.8 ^ab^	70.0 ^ab^	0.49 ^b^
	PA2	126.3	81.1 ^a^	633.3 ^b^	45.2 ^b^	0.66 ^a^
	SEM	6.0	3.7	204.3	6.9	0.03
	Linear	0.518	0.002	0.066	0.027	0.001
	Quadratic	0.811	0.004	0.190	0.081	0.005
Source of variation				*p*-Value		
Breed		0.190	<0.001	<0.001	<0.001	<0.001
Diet		0.638	0.013	0.235	0.124	0.006
Breed × Diet		0.892	0.002	0.432	0.155	0.002

^a–c^ Means within a column with different letters differ significantly (*p* < 0.05). T-Chol, total cholesterol; HDL-C, high-density lipoprotein cholesterol; TG, triglyceride; non-HDL, T-Chol minus HDL-C; HDL/T, ratio of HDL-C to T-Chol. ^1^ RIR = Rhode Island Red; SF = Silky Fowl. ^2^ Cont = basal diet; PA1 = basal diet with 30 mg/kg paprika extract carotenoids; PA2 = basal diet with 60 mg/kg paprika extract carotenoids.

**Table 5:** Effect of storage temperature and breed difference on feeding of paprika extract (PA2 ^1^) on total carotenoid content, yolk color, and pH after 21 days.

Breed ^2^	Temp	T-Caro ^3^	YCFS ^4^	L*	a*	b*	a/b	Albumen pH	Yolk pH
RIR	4 °C	8.67 ^a^	17.0 ^a^	44.7 ^c^	30.6	33.0 ^b^	0.93 ^a^	8.95 ^b^	6.22 ^b^
	25 °C	7.41 ^b^	14.7 ^b^	48.3 ^bc^	28.8	32.6 ^b^	0.96 ^a^	9.51 ^a^	6.67 ^ab^
SF	4 °C	6.34 ^bc^	16.5 ^a^	48.7 ^b^	28.0	36.9 ^ab^	0.77 ^b^	9.40 ^a^	6.43 ^ab^
	25 °C	5.72 ^c^	14.4 ^b^	52.4 ^a^	30.6	43.9 ^a^	0.72 ^b^	9.58 ^a^	6.60 ^a^
Source of variation					*p*-Value				
Breed		<0.001	0.002	<0.001	0.994	<0.001	<0.001	<0.001	0.134
Temp		0.007	<0.001	<0.001	0.826	0.145	0.710	<0.001	0.006
Breed × Temp		0.354	0.693	0.916	0.097	0.092	0.313	<0.001	0.825

^a–c^ Means within a column with different letters differ significantly (*p* < 0.05). ^1^ PA2 = basal diet with 60 mg/kg carotenoid from paprika extract. ^2^ RIR = Rhode Island Red; SF = Silky Fowl. ^3^ Total carotenoid content in egg yolk (mg/100 g yolk). ^4^ Yolk color was determined using the DSM-YCF score (1–16) and was set to 17 when the score exceeded 16.

## Data Availability

The datasets generated for this study are available upon request from the corresponding author.
